# Transforming the management of chronic kidney disease-associated anemia using daprodustat

**DOI:** 10.1097/MS9.0000000000002207

**Published:** 2024-05-20

**Authors:** Ifrah I. Raza, Shaheera Younus, Hiba Azhar, Hareer Fatima, Zainab Anwar, Asma A. Farah, Hussain Sohail Rangwala

**Affiliations:** a Department of Medicine, Jinnah Sindh Medical University, Karachi, Pakistan; b Department of Medicine, East Africa University, Boosaaso, Somalia

The chronic kidney disease (CKD) is characterized as a pathological condition in which the GFR drops below 60 ml/min/1.73 m^2^ ^1^. Over 10% of the general population is affected by chronic kidney disease or CKD, accounting for over 800 million individuals worldwide^2^. Each year, it causes more than 1.1 million deaths globally, according to the WHO^1^. Studies have shown the presence of anemia in patients with CKD and its association with worse prognostic outcomes in terms of extended quality of life and mortality^1^. Chronic kidney disease outcomes and practice patterns study (CKDopps) confirmed that 28–52% of 6766 CKD patients with hemoglobin (Hb) less than 12 g/dl were being followed in France, Germany, Brazil, and the USA^3^. Anemia in CKD is caused mainly by a decrease in erythropoietin (EPO) production in the failing kidney. However, complex pathogenesis includes other causes of anemia, including deficiencies such as iron and vitamins or altered iron hemostasis^4^.

EPO is predominantly produced in the kidneys and EPO gene transcription controls it. An important factor regulating its expression is the hypoxia-inducible factor (HIF) system, the activity of which depends on tissue oxygenation levels. CKD is associated with reduced renal blood flow. It adapts the kidneys to consume less oxygen and subsequently maintains a normal tissue oxygen gradient. As a result, the HIF system is not activated and the EPO gene remains inactive, leading to decreased EPO production in the kidneys^5^.

Furthermore, increased levels of inflammatory mediators in CKD also inhibit hypoxia-induced EPO gene activation. One of these mediators, the acute-phase reactant, also known as hepcidin, plays a significant role in disease pathophysiology^5^. In excess, this iron-regulating peptide causes a decrease in serum iron levels by trapping iron within macrophages and hepatocytes and decreasing intestinal iron absorption, resulting in iron deficiency^6^. Under normal circumstances, serum EPO lowers hepcidin levels, which in turn increases iron absorption and utilization^5^.

For the treatment of anemia in CKD, Epoetin alfa was licensed by the Food and Drug Administration (FDA) in 1989 for the treatment of anemia in CKD, from which novel erythropoiesis-stimulating agents (ESAs) were later developed. ESAs are biological factors that affect EPO receptors and stimulate RBC production. According to the guidelines provided by the kidney disease improving global outcome (KDIGO), the Hb target with ESAs is <11.5 g/dl and variability of even 1 g/dl increases the risk of death by 33%. For this narrow target range, it is difficult to prescribe ESAs to patients with NDD CKD. The drug must be frequently dose adjusted and is associated with an increased risk of MI, thromboembolism, stroke, tumor progression/recurrence, and mortality^7^.

Continuous erythropoietin receptor activator (CERA) has been used as maintenance treatment for anemia in many patients with CKD. Tabata *et al*.^8^ in their prospective, observational, and multicenter study concluded that CERA is safe to be used in anemia in CKD. The drug includes some side effects such as hypertension and anemia; however, it does not cause the development of malignant tumors or pure red cell aplasia.

Ferric citrate, a phosphate binder, is approved as an oral iron supplement for patients with CKD. Increased concentrations of FGF23 are associated with adverse outcomes in patients with iron-deficiency anemia. Ferric citrate reduces circulating FGF23 concentrations, thereby improving serum iron levels. In 2019, Block *et al*. conducted a 36-week trial of ferric citrate versus standard care in 199 patients with CKD. The study concluded that ferric citrate increased Hb levels while decreasing circulating FGF23 levels. However, ferrous citrate causes adverse effects such as discolored stools, diarrhea, and constipation^9^.

Ziltivekimab, an anti-inflammatory monoclonal antibody targeting the IL-6 ligand, was assessed in a phase 2 trial of 264 individuals with CKD and increased CRP levels. The study’s outcome revealed that high CRP levels decreased by 77, 88, and 92% at 7.5, 15, and 30 mg, respectively, 12 weeks later^10^. Thus, decreasing inflammation can be a fundamental change in the treatment of CKD patients.

On 1 February 2023, the FDA approved daprodustat, an orally administered hypoxia-inducible factor prolyl hydroxylase (HIF-PH) inhibitor for the treatment of anemia in patients with CKD on dialysis^11^. Hypoxia in CKD results in activation of the HIF system, which leads to the accumulation and translocation of HIF1-α to the cell nuclei, where it binds to HIF1-β. The HIF1 α-β heterodimer activates EPO gene expression by binding to DNA sequences called hypoxia response elements (HRE)^5^. This promotes an erythropoietic response and upregulates iron transport by decreasing the hepcidin levels. At adequate oxygen levels, HIF-PH regulatory enzyme degrades HIF-α. Daprodustat inhibits this enzyme, thereby increasing HIF-α, decreasing hepcidin, increasing EPO, and upregulating iron transport^5^ (Fig. 1).

**Figure 1:**
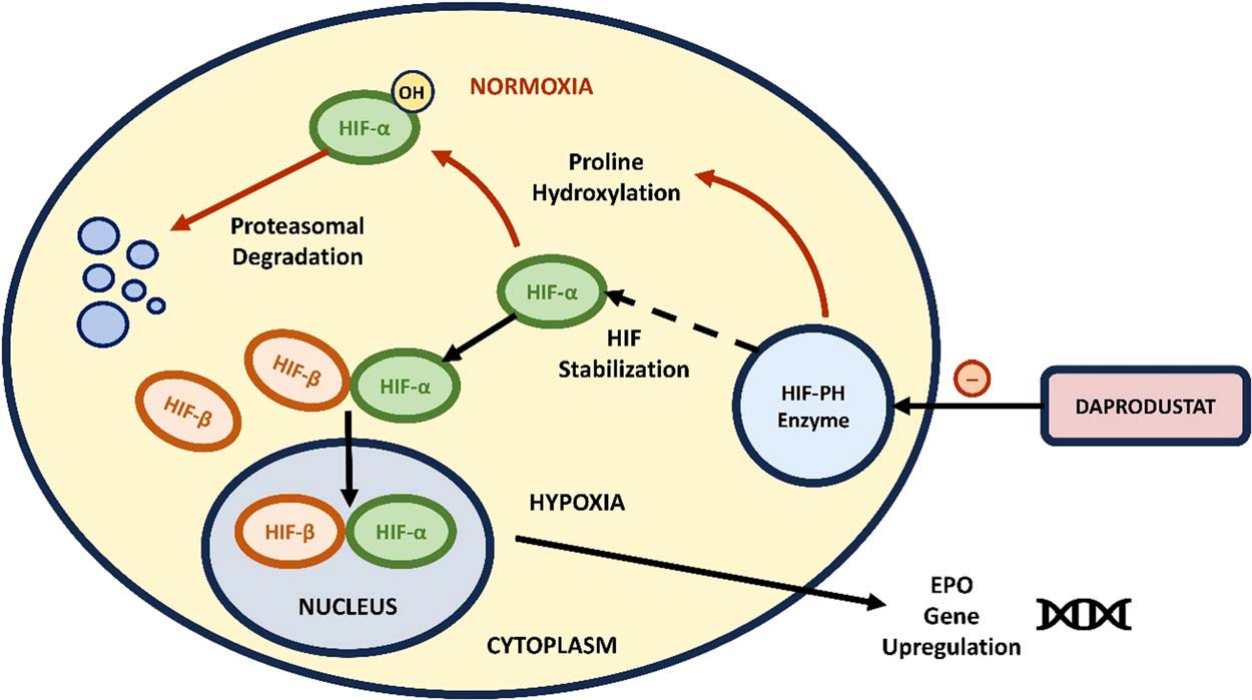
Mechanism of action of daprodustat. Under normal conditions, rapid degradation of HIF1-α occurs through the action of the HIF-PH enzyme (as indicated by the red arrows). However, during hypoxia, the HIF-PH enzyme is suppressed, which leads to upregulation of the EPO gene by the formation of the HIF1 α-β heterodimer (as indicated by the black arrows). Daprodustat has direct inhibitory effects on the HIF-PH enzyme^12^.

Randomized, open-label ASCEND phase 3 clinical trials analyzed daprodustat and became the basis for its FDA approval. Two pivotal trials, ASCEND-D and ASCEND-ND, compared daprodustat with treatments injected with ESA (epoetin alfa or darbepoetin alfa). The efficacy of the drug was determined by the mean change in Hb level from baseline to weeks 28 through 52 and the safety of the first occurrence of a major adverse cardiovascular event (MACE). In ASCEND-D, 2964 patients were randomized. The mean change in Hb level from baseline was 0.28±0.02 g per deciliter in the daprodustat group and 0.10±0.02 g per deciliter in the ESA group (a difference of 0.18 g per deciliter). During an average follow-up of 2.5 years, MACE occurred in 25.2% (374 out of 1487) of patients in the daprodustat group and 26.7% (394 out of 1477) of patients in the ESA group^13^. In ASCEND-ND, 3872 patients were randomized to receive either daprodustat or darbepoetin alfa. The mean change in Hb level from baseline was 0.74±0.02 g per deciliter in the daprodustat group and 0.66±0.02 g per deciliter in the darbepoetin alfa group (a difference of 0.08 g per deciliter). During an average follow-up of 1.9 years, MACE occurred in 19.5% (378 out of 1937) of patients in the daprodustat group and 19.2% (371 out of 1935) of patients in the darbepoetin alfa group^14^. Both trials found that daprodustat was noninferior to ESAs in terms of efficacy and safety.

CYP2C8 metabolizes daprodustat. Dose adjustment is required for hepatic impairment when co-administered with CYP2C8 inhibitors^15^. The most common adverse effects include nausea, pain in the extremities, diarrhea, dyspepsia, upper respiratory tract infections, and fatigue. A randomized study found that 8% of patients experienced SAEs such as congestive heart failure, arrhythmias, hyponatremia, and COPD. Black-box warnings include an increased risk of thrombotic vascular events, MI, and stroke^12^. In 2023, Sackeyfio *et al*. performed an analysis to determine the cost-effectiveness of daprodustat compared to recombinant human EPO for treating anemia in CKD. The study reported that daprodustat is not only less costly compared to Darbepoetin alfa and Epoetin alfa but also enhances the quality-adjusted life years (QALYs). Its cost-effectiveness is further influenced by the decreased need for administration and eliminated requirement of cold chain storage^16^.

Approval for this drug has expanded the options for the treatment of anemia in CKD, especially in patients who are hyporesponsive to ESAs. Oral therapy to improve anemia in end-stage renal disease can be beneficial, particularly in terms of cost and cannula-related complications. However, to become the preferred medication in patients responsive to ESAs, daprodustat needs to be well tolerated over long periods of usage and proven to be superior to ESAs in improving clinical outcomes. Further research is required to determine the impact of long-term usage on clinical outcomes and associated adverse effect profiles.

## Ethical approval

Ethical approval was not necessary for this editorial.

## Consent

Patient consent: N/A. Parental consent: N/A. The editorial is derived from information available online. It does not include patients or volunteers as subjects. Thus, consent is not applicable.

## Sources of funding

The authors received no extramural funding for the study.

## Author contribution

I.I.R. and H.A.: conceptualization; S.Y., I.I.R., H.F., H.S.R., A.A.F., Z.A., and H.A.: literature and drafting of the manuscript; H.F. and S.Y.: editing and supervision. All authors have read and agreed to the final version of the manuscript. H.S.R.: performed the task of reviewing and editing the editorial.

## Conflicts of interest disclosure

The authors declare no potential conflicts of interest concerning the research, authorship, and/or publication of this article.

## Research registration unique identifying number (UIN)

1. Name of the registry: not applicable.
2. Unique Identifying number or registration ID: not applicable.
3. Hyperlink to your specific registration (must be publicly accessible and will be checked): not applicable.

## Guarantor

All authors take responsibility for the work, access to data and decision to publish.

## Data availability statement

Not applicable.

## Provenance and peer review

Not commissioned, externally peer-reviewed.
